# Short-term complications following distal humerus open reduction and internal fixation

**DOI:** 10.1016/j.jseint.2026.101620

**Published:** 2026-01-14

**Authors:** Sashrik Sribhashyam, Grayson M. Talaski, Shahabeddin Yazdanpanah, Carl Edge, Matthew S. Smith, Andrew S. Cuthbert, Jennifer L. Vanderbeck

**Affiliations:** a Virginia Commonwealth University School of Medicine, Richmond, VA, USA; b Department of Orthopedics and Rehabilitation, University of Iowa, Iowa City, IA, USA; c College of Medicine, Northeast Ohio Medical University, Rootstown, OH, USA; d Department of Orthopaedic Surgery, Virginia Commonwealth University Health System, Richmond, VA, USA

**Keywords:** Distal, Humerus, Open reduction and internal fixation, ORIF, Complication, Database

## Abstract

**Background:**

Distal humerus fractures (DHFs) account for around 2% of all adult fractures. Since nonoperative strategies often lead to loss of motion and disability from prolonged immobilization, open reduction and internal fixation (ORIF) is a commonly employed first-line treatment for reconstructable DHFs and can yield satisfactory outcomes. Surgical recovery, however, is not without complications, with reported rates up to 30%. Prior studies often report ORIF in pooled or comparative settings, leaving a gap in isolated ORIF outcomes. Therefore, this study aims to analyze short-term postoperative complications following DHF ORIF.

**Methods:**

The American College of Surgeons National Surgery Quality Improvement Program database was queried using the Current Procedural Terminology code 24579. Patients with missing relevant variables were excluded. Postoperative outcomes included surgical site infection, wound dehiscence, return to the operating room (ROR), and any adverse event (AAE), among others. Continuous variables were reported as mean (standard deviation) and binary variables as number (%). Multivariate logistic regression with Bonferroni correction was used to model associated risk factors. Additionally, threshold analysis was applied to operative time for modeling complication risk.

**Results:**

A total of 833 patients were identified (mean age = 53.9 ± 19.9; 71.8% female; 70.1% white; 68.3% outpatient; 50.1% American Society of Anesthesiologists class 2). Cohort comorbidities included smoking (18%) and diabetes (8.9%; 5.3% non-insulin dependent; 3.6% insulin-dependent). Overall, complication rates were low, with AAE occurring at 5.2% within 30 days. Surgical site infection, ROR, and wound dehiscence were all below 2%. Age (odds ratio [OR] = 1.03), operative time (OR = 1.01), hospital length of stay (OR = 1.2), and smoking (OR = 2.07) significantly increased AAE risk. A 97.1-minute significant operative time threshold was calculated, with complication rates of 7.8% and 1.4% above and below this cutoff, respectively (OR = 5.62).

**Discussion:**

DHF ORIF demonstrates low short-term complication rates. However, factors such as increased age and smoking elicit reasonable operative concerns. Prolonging operative time was significantly associated with increased risks for AAE, the highest of which was observed beyond a 97-minute threshold for operative time. Targeted counseling is recommended, and future studies are warranted to further granularize outcomes.

Distal humerus fractures (DHFs) are a common injury, representing around 2% of all adult fractures and half of the fractures that occur around the elbow.^2^^,^^4^^,^^5^^,^^7^^,^^12^^,^^16^^,^^20^ These injuries often involve articular fragments and occur in a region of complex anatomy, which together pose significant challenges for surgical repair.^4^^,^^11^^,^^12^^,^^15^ In older patients, osteoporotic bone can further complicate fixation.^2^^,^^4^^,^^10^, ^11^, ^12^^,^^15^^,^^17^ In addition to patient factors, injury severity and fracture pattern, particularly comminuted intra-articular Arbeitsgemeinschaft für Osteosynthesefragen/Orthopaedic Trauma Association (AO/OTA) type C injuries and open fractures, are associated with higher rates of complication after open reduction and internal fixation (ORIF), including infection, nonunion problems, and heterotopic ossification.^6^^,^^13^^,^^21^ ORIF is a commonly employed first-line treatment for reconstructable DHFs and can yield satisfactory outcomes, but various factors can complicate the recovery.^2^^,^^5^^,^^10^ The overall complication rate for DHFs, encompassing primary (trauma-associated), intraoperative, and postoperative issues, can be as high as 30%.^11^^,^^15^ Nevertheless, surgical management is typically favored over nonoperative treatment in patients who can tolerate surgery, as prolonged immobilization without surgery often leads to stiffness and poor functional outcomes.^5^^,^^12^^,^^15^, ^16^, ^17^

Numerous studies have examined risk factors and outcomes in DHF surgery, highlighting patient and perioperative factors that influence postoperative complications. For example, obesity has been linked to higher rates of 90-day local and systemic complications following DHF fixation.^20^ Patient frailty has also emerged as a critical predictor: using a five-item modified frailty index, Saltzman et al demonstrated that more frail patients had significantly increased rates of 30-day complications, hospital readmissions, and reoperations after surgical management of DHFs.^17^ In the context of geriatric patients, debate remains about the optimal surgical construct. Some comparative studies report similar short-term complication rates between ORIF and total elbow arthroplasty (TEA) in elderly cohorts, whereas others have noted specific differences.^10^^,^^18^ Most notably, a recent propensity-matched analysis found that TEA carries a higher risk of postoperative blood transfusion compared with ORIF despite otherwise comparable overall 30-day complication rates.^9^^,^^18^ Certain complications are uniquely associated with ORIF. For instance, a large registry study reported that intraoperative manipulation of the ulnar nerve during ORIF was linked to an increased incidence of postoperative ulnar neuropathy.^4^ Although these investigations emphasize important risk factors and inform treatment considerations, their scope has often been limited. Many analyses either pooled different surgical modalities or focused on specific subpopulations, and a comprehensive, procedure-specific evaluation of ORIF outcomes in a broad patient cohort has been lacking.^7^^,^^12^^,^^17^^,^^18^

Given this gap in the literature, the present study aims to provide a robust analysis of the types and rates of 30-day short-term postoperative complications following ORIF for DHFs in a large national patient sample. By isolating ORIF cases and examining associated risk factors, this investigation addresses the need for an up-to-date, ORIF-specific outcomes assessment. The goal is to improve preoperative patient counseling and guide strategies for minimizing complications in the management of DHFs.

## Methods

### Database

In this retrospective database study, relevant cases of ORIF for DHFs were queried in the American College of Surgeons National Surgery Quality Improvement Program (NSQIP) database. The NSQIP collects 30-day surgical data prospectively from participating institutions. These data include preoperative characteristics, intraoperative variables, and rates of complications. The most recent NSQIP database iteration in 2023 included 994,313 cases across 676 participating institutions and includes 274 unique variables.^1^ Random audits of participating sites found inter-rater disagreement rates of approximately 2%, which demonstrates excellent reliability.^3^^,^^19^

### Patient cohort

In this study, patients over 18 years old who received ORIF for DHFs were identified using the Current Procedural Terminology code 24579 from 2010 to 2023. Criteria for exclusion included unknown or null values for sex, body mass index, functional status, American Society of Anesthesiologists classification, operative time, anesthesia technique, or total length of hospital stay.

### Outcome metrics

Postoperative outcome data were collected for each patient and included death within 30 days, surgical site infection (SSI), wound dehiscence, acute renal failure requiring dialysis, sepsis, pulmonary embolism, myocardial infarction, cardiac arrest, unplanned intubation, postoperative transfusion, deep vein thrombosis, pneumonia, urinary tract infection, return to the operating room (ROR), and extended length of stay (LOS). Extended LOS was defined as any LOS greater than the mean LOS. Any adverse event (AAE) was defined as any occurrence of SSI, wound dehiscence, sepsis, unplanned intubation, postoperative transfusion, pneumonia, deep vein thrombosis, pulmonary embolism, urinary tract infection, stroke, cardiac arrest, myocardial infarction, ROR, and death within 30 days.

### Statistical analysis

Statistical analyses were conducted in RStudio (version 2023.06.1 + 524, R Foundation for Statistical Computing, Vienna, Austria). Continuous variables are summarized as means and standard deviations, while categorical variables are summarized as counts and percentages. Multivariable logistic regression models quantified associations between comorbidities and outcomes, yielding odds ratios (ORs) with 95% confidence intervals. Highly collinear predictors were excluded after an ad hoc screening procedure to prevent model redundancy. To control the family-wise error rate due to multiple comparisons, a Bonferroni correction was used.

A systematic threshold search identified the operative time cutoff most strongly linked to complications. Multiple candidate thresholds were tested, and the value producing the highest adjusted OR that remained statistically significant was selected. To maintain clinical relevance, the maximum operative time was cut off at 300 minutes. Final multivariable models evaluated two outcomes: AAE and SSI. Statistical significance was defined as *P* < .05 throughout.

## Results

### Patient demographics

A total of 833 patients were identified within the American College of Surgeons-NSQIP database. The average age of these patients was 53.9 (standard deviation [SD] = 19.9), with an average body mass index of 29.1 (SD = 7.47). Our cohort was primarily female (71.8%), outpatient (68.3%), American Society of Anesthesiologists class 2 (50.1%), and white (70.1%). Furthermore, 18% of our cohort identified as smokers, and 8.9% were diabetic (5.3% non-insulin-dependent, 3.6% insulin-dependent) (Table I).

**Table I:** Demographic information for patients who received ORIF for distal humerus fractures between 2010 and 2023.

Metric	Mean (SD) or n (%)
Patients	833
Age (yr)	53.9 (19.9)
Body mass index (kg/m^2^)	29.1 (7.47)
Sex	
Male	235 (28.2%)
Female	598 (71.8%)
Operative time (min)	128 (72.1)
Total hospital LOS (d)	1.28 (2.51)
Inpatient/Outpatient	
Inpatient	264 (31.7%)
Outpatient	569 (68.3%)
Smoker	
Yes	150 (18.0%)
No	683 (82.0%)
Time from admission to operation	0.353 (1.37)
Steroid use	
Yes	32 (3.8%)
No	801 (96.2%)
Bleeding disorder	
Yes	27 (3.2%)
No	806 (96.8%)
Diabetes	
Insulin	30 (3.6%)
Noninsulin	44 (5.3%)
No	759 (91.1%)
COPD history	
Yes	25 (3.0%)
No	808 (97.0%)
CHF history	
Yes	5 (0.6%)
No	828 (99.4%)
Functional status	
Independent	806 (96.8%)
Partially dependent	23 (2.8%)
Totally dependent	4 (0.5%)
Race	
White	584 (70.1%)
Black or African American	32 (3.8%)
Asian	25 (3.0%)
Other/Unknown	192 (23.0%)
ASA classification	
1 – no disturb	128 (15.4%)
2 – mild disturb	417 (50.1%)
3 – severe disturb	264 (31.7%)
4 – life threat	23 (2.8%)
5 - moribund	1 (0.1%)

*ORIF*, open reduction and internal fixation; *LOS*, length of stay; *ASA*, American Society of Anesthesiologists; *COPD*, chronic obstructive pulmonary disease; *CHF*, congestive heart failure; *SD*, standard deviation.

### Complication rates

Overall, complication rates were low across all studied metrics, with the risk of developing any 30-day postoperative adverse event being 5.2%. Commonly concerning metrics such as SSI, ROR, and wound dehiscence were all below 2%. The rate of unplanned intraoperative transfusion was 1.6%, and all remaining complication rates were below 1%. Extended LOS, while not labeled as a traditional complication due to conflicting definitions across the literature, had a rate of 9% (Table II).

**Table II:** Complication rates in patients who underwent ORIF for distal humerus fractures between 2010 and 2023.

Metric	Mean (SD) or n (%)
Dialysis required	1 (0.1%)
Blood transfusion	2 (0.2%)
Surgical site infection	6 (0.7%)
Death	4 (0.5%)
Intubation required	1 (0.1%)
Transfusion	13 (1.6%)
Pneumonia	1 (0.1%)
Urinary tract infection	3 (0.4%)
Deep vein thrombosis	3 (0.4%)
Return to operating room	12 (1.4%)
Any adverse event	43 (5.2%)
Wound dehiscence	2 (0.2%)
Pulmonary embolism	2 (0.2%)
CVA/Stroke	2 (0.2%)
Myocardial infarction	2 (0.2%)
Extended LOS	75 (9.0%)

*ORIF*, open reduction and internal fixation; *LOS*, length of stay; *CVA*, cerebrovascular accident; *SD*, standard deviation.

### Odds ratio (any adverse event)

Regarding multivariate OR logistic regression analysis for risk of AAE, numerous factors significantly impacted the risk of complications. Age (OR = 1.03 [1.01-1.05], *P* = .03), operative time (OR = 1.01 [1.00-1.01], *P* = .001), smoking history (OR = 2.07 [1.05-4.07], *P* = .042), and hospital LOS (OR = 1.2 [1.08-1.34], *P* = .001) all increased the risk of postoperative adverse events (Table III).

**Table III:** Multivariate odds ratio logistic regression for risk of any adverse event.

Variable	Odds ratio	95% CI	*P* value	Adjusted *P* value
Age (yr)	1.03	(1.01-1.05)	.01	.03
BMI (kg/m^2^)	0.96	(0.92-1.01)	.06	.172
Operative time (min)	1.01	(1.00-1.01)	<.001	.001
Total hospital LOS (d)	1.2	(1.08-1.34)	<.001	.001
Time from admission to operation	0.86	(0.61-1.05)	.26	.33
History of smoking	2.07	(1.05-4.07)	.035	.042
History of COPD	3.76	(1.23-11.49)	.020	.053
Hypertension medication usage	1.65	(0.89-3.09)	.115	.172
History of steroid use	1.46	(0.32-4.93)	.58	.65
History of CHF	12.81	(2.08-78.81)	.006	.018
Bleeding disorder	2.40	(0.69-8.30)	.168	.235
Intraoperative transfusion	18.81	(1.16-305.99)	.039	.082
Diabetes				
Insulin-dependent	1.33	(0.31-5.77)	.704	.778
Non-insulin-dependent	1.37	(0.41-4.62)	.610	.771

*BMI*, body mass index; *LOS*, length of stay; *COPD*, chronic obstructive pulmonary disease; *CHF*, congestive heart failure; *CI*, confidence interval.

### Odds ratio (surgical site infection)

Regarding multivariate OR logistic regression calculations, the risk of developing postoperative SSI after ORIF of DHFs was not significantly increased in any studied metrics. However, with the overall low rate of SSI, many comorbidities were unable to be assessed (Table IV).

**Table IV:** Multivariate odds ratio logistic regression for risk of developing a surgical site infection (SSI).

Variable	Odds ratio	95% CI	*P* value	Adjusted *P* value
Age (yr)	0.98	(0.94-1.02)	.29	1.00
BMI (kg/m^2^)	0.96	(0.86-1.08)	.51	1.00
Operative time (min)	1.00	(0.99-1.01)	.53	1.00
Total hospital LOS (d)	0.26	(0.01-1.05)	.18	1.00
Time from admission to operation	4.22	(0.19-91.56)	.20	1.00
History of smoking	9.34	(1.70-51.49)	.010	.22
Hypertension medication usage	0.44	(0.05-3.82)	.46	1.00
History of steroid use	5.14	(0.58-45.34)	.14	.98

*BMI*, body mass index; *LOS*, length of stay; *CI*, confidence interval.

### Operative time threshold

**Figure 1:**
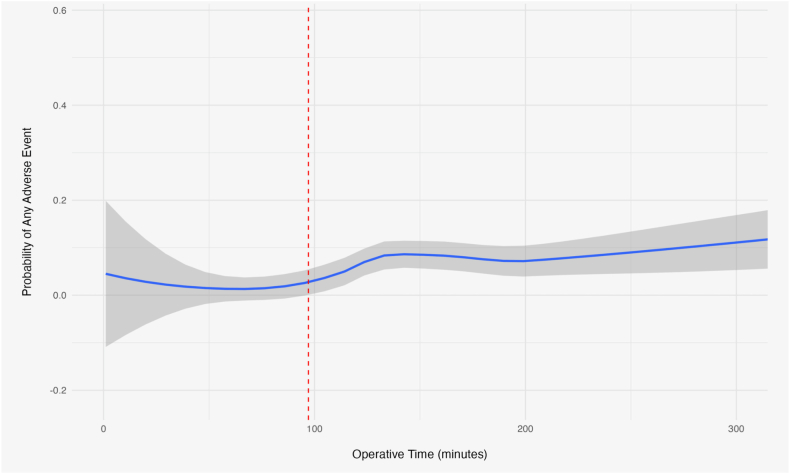
Relationship between operative time (minutes) and the probability of adverse events.

To further assess the increased risk of developing AAE with increased operating time, operative time threshold calculations were performed. An operative time cutoff value of 97.1 minutes was calculated, with patients above and below this threshold demonstrating complication rates of 7.8% and 1.4%, respectively (OR = 5.62 [2.38-16.5], *P* < .001). The relationship between operative time and the likelihood of experiencing an adverse postoperative event is shown (Fig. 1).

## Discussion

The present analysis of 833 distal humerus ORIF cases demonstrates short-term morbidity after isolated plate fixation is relatively low. In our cohort, only 5.2% of patients experienced AAE within 30 days of surgery, and the incidence of individual wound complications (such as SSI or dehiscence) remained below 2%. Nevertheless, multivariate modeling identified several meaningful and largely modifiable risk factors. Each decade of age conferred roughly a 30% relative increase in complications. Longer operative duration was also associated with higher complication risk: every extra minute in the operating room conferred an incremental increase, and we identified an inflection point at approximately 97 minutes beyond which the complication rate rose fivefold (7.8% vs. 1.4%). Prolonged hospital LOS was another strong predictor of complications, and current smoking status more than doubled the odds of a postoperative adverse outcome.

Our 5.2% composite complication rate is lower than the 8%-12% reported in elderly only cohorts that compared ORIF with TEA using the same NSQIP platform and the 6.9% observed in a recent propensity-matched analysis of patients in their mid-70s.^7^^,^^10^, ^11^, ^12^^,^^18^ It is dramatically below the pooled 32.6% rate described in the 2014 meta-analysis of historical series, where minor events dominated the tally.^7^ Several factors likely explain this favorable distinction. First, our patient population was substantially younger (mean age 54 years) and generally healthier than those in many previous series that concentrated on patients in their 80s.^12^^,^^17^ Notably, because frail elderly patients with DHFs are now often managed with primary TEA instead of ORIF, our ORIF-only sample inherently excluded some of the highest-risk individuals.^11^^,^^12^^,^^17^ Large U.S. database studies suggest that TEA constitutes roughly 10 to 15% of operative management for geriatric distal humeral fractures, increasing from 5.1% in 2002 to 13% in 2012 in a Nationwide Inpatient Sample, with more recent NSQIP data from 2012 to 2021 showing TEA in 11.3% of surgically treated DHF cases.^14^^,^^18^ Second, two-thirds of cases were performed as outpatient procedures, suggesting a shift toward less physiologically frail patients and more streamlined perioperative pathways.^17^ Finally, advances in precontoured locking implants and elbow approaches over the last decade may have shortened surgical exposure and limited soft tissue insult.^5^^,^^18^

Within our cohort, increasing patient age was independently associated with higher complication risk, which aligns with the findings of Sandoval et al that age was the only significant risk factor for 30-day adverse events when ORIF and TEA outcomes were analyzed together.^18^ This emphasizes that advanced age contributes to postoperative vulnerability, but chronological age alone should not be the sole criterion in treatment decisions. Careful geriatric assessment is warranted to distinguish fit elderly patients from those with limited physiological reserve.^12^^,^^17^ Although age contributed to risk, frailty appears more informative for short-term stratification than age alone. Saltzman et al demonstrated that DHF patients with a modified frailty index greater than or equal to 2 experienced nearly a fivefold higher complication rate compared to their more robust counterparts.^17^ Consistently, we found that each additional day of hospitalization, a postoperative marker of resource utilization and clinical complexity, was associated with higher odds of an adverse event. These observations support the concept that a patient's overall frailty status may better capture short-term risk than chronological age alone.^17^

While prior database studies described longer mean times for TEA than ORIF, none defined a complication threshold for ORIF specifically.^10^^,^^11^^,^^18^ The 97-minute cutoff we identified offers a practical benchmark for teams to monitor intraoperative efficiency. Cases exceeding this duration had a markedly higher complication rate, suggesting that prolonged operative time serves as a proxy for increased case complexity or technical difficulty. Fracture severity and soft tissue injury likely contribute to this association. In a population-based cohort of 320 adult DHFs, Robinson et al reported that union complications were more common after high-energy mechanisms, fractures, and nonoperative treatment.^16^ Infection, myositis ossificans, and implant-related complications were higher following operative treatment of AO/OTA type C fractures compared with type A and B injuries.^16^ In addition, a meta-analysis of intra-articular distal humeral fractures treated with ORIF reported an overall complication rate of 53% and a reoperation rate of 21%, highlighting the inherent complication burden of more complex articular patterns.^21^ Because NSQIP lacks fracture-level variables like AO/OTA subtype, comminution, intra-articular involvement, and open fracture status, prolonged operative time in our cohort likely reflects a combination of injury, complexity, and patient factors rather than surgical efficiency alone.^16^^,^^21^ Lengthy surgeries often involve complex articular reconstructions, extensive soft tissue dissection, or other intraoperative challenges.^2^^,^^4^^,^^15^ Fortunately, many factors (insufficient preoperative planning, suboptimal exposure, or lack of experienced assistance) contributing to such delays are addressable. Meticulous preoperative preparation and having skilled support available may help keep operative times within a safer range.^2^^,^^5^

Patient health habits also played a role in outcomes. Active tobacco use was an independent predictor of complications in our analysis (OR = 2.07). Although smoking history is not often highlighted in DHF-specific literature, the deleterious effects of smoking on fracture healing and infection risk are well-documented in orthopedic surgery.^8^ This finding reinforces the importance of smoking cessation initiatives as part of preoperative optimization for elbow fracture patients. By contrast, obesity did not significantly influence 30-day complication rates in our cohort. This result differs from that of Werner et al who observed substantially higher 90-day complication odds in obese patients undergoing distal humerus ORIF.^20^ The discrepancy may be explained by our shorter follow-up window, so certain obesity-related problems (such as wound breakdown or deep infection) might manifest beyond the initial 30-day postoperative period.

Overall, our data suggest that short-term postoperative complications after ORIF of DHFs have improved and are now comparable to those reported for TEA within similar time frames.^10^^,^^18^ Nevertheless, surgeons should remain vigilant for patients with high-risk features, including advanced age or frailty, longer operative times, active smoking, and early postoperative setbacks such as prolonged hospitalization.^12^^,^^17^^,^^20^ Recognizing these risks allows for targeted interventions such as meticulous preoperative templating, efforts to optimize intraoperative efficiency, and aggressive perioperative risk factor management (eg, smoking cessation) which may further reduce complication rates.^2^^,^^5^^,^^20^

Strengths include the largest contemporary ORIF-only sample to date, a 13-year study window, and the novel threshold analysis for operative duration. Limitations mirror those inherent to NSQIP-based work: potential coding errors, absence of fracture-pattern detail, lack of surgeon or implant variables, inability to reliably capture nerve injuries, and surveillance restricted to 30 days.^4^^,^^10^^,^^12^^,^^17^^,^^18^^,^^20^ These are limitations acknowledged in prior database studies of elbow trauma.^4^^,^^10^^,^^12^^,^^17^^,^^18^^,^^20^ Our younger, outpatient-heavy cohort may also limit generalizability to frailer populations.^10^^,^^12^^,^^17^^,^^20^ Finally, low event counts for specific complications limited multivariable modeling power, particularly for SSI.^10^^,^^12^^,^^16^^,^^17^ Prospective multicenter registries capturing fracture morphology, implant constructs, standardized nerve injury reporting, and functional scores beyond 30 days are needed to validate these findings and to explore whether the 97-minute operative-time benchmark translates to long-term outcomes.^4^^,^^7^^,^^10^, ^11^, ^12^^,^^17^^,^^18^ Incorporating frailty indices into routine preoperative assessment and studying targeted optimization protocols represent other high-yield avenues.^17^

## Conclusion

This short-term NSQIP database study demonstrated positive complication-related outcomes following ORIF for DHFs. Notably, classical wound-related complications were relatively uncommon. Though expected, increasing age and prolonging operative time were significantly associated with increased risks for AAE, the highest of which was observed beyond a 97-minute threshold for operative time. Furthermore, patients with a history of smoking had an increased risk of developing AAE. Targeted patient counseling is critical to curbing AAEs in such cohorts. Though inherently limited due to the constraints of this NSQIP database, the present study's overall short-term outcomes of DHF ORIF demonstrated accepted results with all complication rates near or below those described in prior literature.

## Disclaimers

Funding: No funding was disclosed by the authors.

Conflicts of interest: The authors, their immediate families, and any research foundations with which they are affiliated have not received any financial payments or other benefits from any commercial entity related to the subject of this article.
